# Rare Mandibular Surgical Ciliated Cysts: First Case Reported in a Brazilian

**DOI:** 10.1055/s-0044-1795077

**Published:** 2024-12-30

**Authors:** Gabriela Lopes-Santos, Hugo Nary Filho, Denise Tostes Oliveira

**Affiliations:** 1 Department of Surgery, Stomatology, Pathology, and Radiology, Area of Pathology, Bauru School of Dentistry, University of São Paulo, Bauru, São Paulo, Brazil; 2 Hugo Nary Institute, Bauru, São Paulo, Brazil

**Keywords:** surgical ciliated cyst, postoperative maxillary cyst, orthognathic surgery, mandibular

## Abstract

This reported case represents the first well-documented mandibular surgical ciliated cyst, following orthognathic surgery consisting of a combination of Le Fort I bimaxillary and sagittal osteotomy concomitantly with genioplasty, reported in a Brazilian patient. A case of 43-year-old female presenting a mandibular surgical ciliated cyst after 16 years of an orthognathic surgery, consisting of a combination of Le Fort I osteotomy and genioplasty, is reported. The cystic lesion was enucleated, and the histopathological analysis showed a cystic cavity lined by pseudostratified columnar respiratory-type epithelium presenting ciliated and mucous cells supported by fibrous connective tissue without inflammation. The diagnosis established was a mandibular surgical cilited cyst. Then, for diagnosis of mandibular surgical ciliated cyst is essential the association of clinical/imagological and histopathological features with the patient' past history showing evidence of previous surgery in the mandible concomitant with the maxilla.

## Introduction

The surgical ciliated cyst also referred as postoperative maxillary cyst, ectopic ciliated cyst, respiratory cyst, or implantation cyst, occurs due the entrapment of fragments of respiratory epithelium into a wound following maxillary sinus surgery or facial trauma. 1 2 3 4

Although primarily linked to maxillary sinus surgery, a similar surgical ciliated cyst was reported within the mandible by Nastri and Hookey, 5 after surgery from a nasal osseocartilaginous graft used for chin augmentation. Currently, the mandibular surgical ciliated cyst has been considered a rare complication following orthognathic surgery produced by implantation of sinus epithelium into a mandibular wound during simultaneous surgery on the maxilla. 1 4 6 7

A recent systematic review of mandibular surgical ciliated cyst confirmed that only 16 cases have been published in the English literature 6 and no case has been reported on a Brazilian population. Most of the patients developed the mandibular surgical ciliated cyst following concomitant genioplasty with rhinoplasty or bimaxillary osteotomy surgery without genioplasty. 6 To our knowledge, this reported case represents the first well-documented mandibular surgical ciliated cyst, following orthognathic surgery consisting of a combination of Le Fort I bimaxillary and sagittal osteotomy concomitantly with genioplasty, reported in a Brazilian patient.

## Case Report

**Fig. 1:**
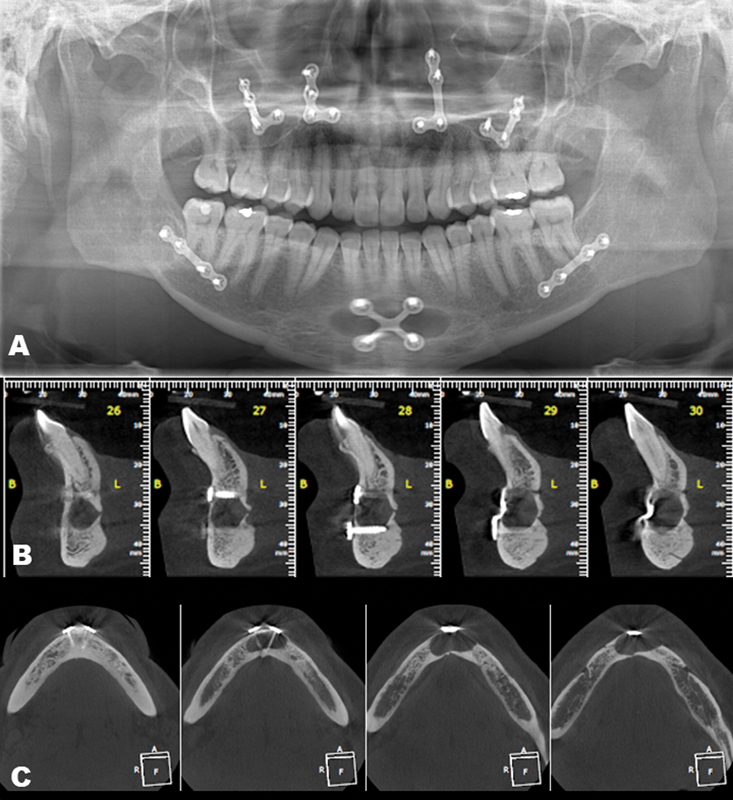
Imaginological images of mandibular surgical ciliated cyst. ( **A** ) Panoramic radiograph demonstrates rigid fixation accessory in the maxilla and mandible. A well-circumscribed radiolucent lesion is also observed close to the apex of teeth 42 to 32. In cone beam computed tomography sagittal ( **B** ) and axial ( **C** ) reconstructions, a hypodense image is noted, closely associated with rigid fixation material.

**Fig. 2:**
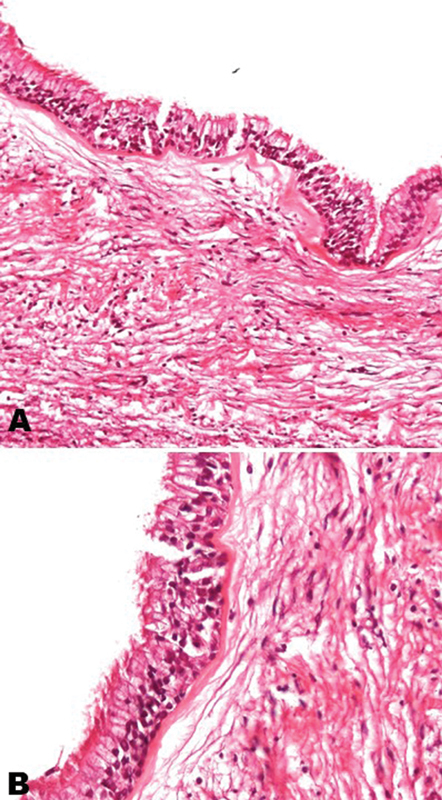
Photomicrography of mandibular surgical ciliated cyst. ( **A** ) Cystic cavity covered by pseudostratified epithelium ciliated and with cells mucous and fibrous capsule with absence of inflammation and in ( **B** ) detail of the respiratory epithelium found in the cyst.

A 43-year-old female was referred for treatment of mandibular intraosseous lesion causing swelling and pain of the chin region. Sixteen years earlier, the patient underwent orthognathic surgery consisting of a combination of Le Fort I bimaxillary and sagittal osteotomy with rigid internal fixation concomitantly with genioplasty. Her current medical history was unremarkable and no postoperative complication following orthognathic surgery was reported. The panoramic radiograph demonstrates rigid fixation accessory in the maxilla and mandible ( Fig. 1A ). The cone beam computed tomography was performed and revealed a well-defined oval and hypodense intraosseous lesion in the anterior mandibular region ( Fig. 1B ), extending from the left to the right canine, intimately involving the internal rigid fixation material. There was no perforation of the labial and lingual mandibular cortical bones or association of the lesion with the apices of the anterior mandibular teeth ( Fig. 1C ). The presumptive clinical diagnosis was odontogenic cyst. The enucleation of the lesion was performed and the surgical specimen sent for histopathological analysis. Microscopic examination showed a cystic cavity lined by pseudostratified columnar respiratory-type epithelium ( Fig. 2A ), presenting ciliated and mucous cells supported by fibrous connective tissue without inflammation ( Fig. 2B ). The final diagnosis established was mandibular ciliated surgical cyst. In the 1-year of follow-up, there was no evidence of recurrence.

## Discussion

Although there are very little published mandibular surgical ciliated cysts, the lesion has been considered one of the unusual or rare complications following orthognathic surgery, mainly involving simultaneous Le Fort I osteotomy and genioplasty. 3 4 5 6 7 8 9 In the present case reported, the patient had undergone an orthognathic surgery, consisting of a combination of Le Fort I bimaxillary and sagittal osteotomy concomitantly with genioplasty, 16 years earlier, confirming that the patient who developed mandibular surgical ciliated cyst may remain asymptomatic for a long period. According to one recent systematic literature review performed by Brisset et al, 6 the mandibular surgical ciliated cyst was most frequent in the anterior mandible and developed within 2 to 56 years with the median latency period of 17 years, a finding closely similar to that observed in our patient.

The ciliated surgical cyst does not present specific and conclusive imaging characteristics for its identification. 1 8 9 However, it generally exhibits well-defined characteristics and does not destroy local teeth and bone. 6 9 Therefore, the use of rigid fixation plates in the maxillary region aids in forming diagnostic hypotheses. In our clinical case, we observed a slight cortical bone resorption without perforation. It is worth noting that the lesion is closely associated with the site of rigid fixation plate implantation.

The etiopathogenic hypotheses for ciliated surgical cysts in the mandible include specific variations and additional considerations due to the anatomy and type of surgical procedures performed in this region. 5 6 During surgical procedures, such as orthognathic surgeries involving the maxillary sinus, fragments of pre-existing respiratory mucosal epithelium may be introduced into the mandibular bone. 1 3 7 9 This implanted epithelium can proliferate and form a cyst. 5 6 Identifying the precise etiology can be challenging, and often a combination of factors may be involved. 6

Typically, as described in the present case, the mandibular surgical ciliated cyst is lined by a pseudostratified columnar epithelium with ciliated and mucous cells without inflammation in the fibrous capsule. These respiratory-type epithelium can be found as metaplastic change of the lining epithelium of the radicular, glandular odontogenic cyst and the dentigerous cyst. 1 9 Although ciliated cells in association with mucous cells may be found in the epithelium lining of radicular cysts, the criterion for its diagnosis is its association with a tooth presenting a nonvital pulp. 1 Additionally, the diagnosis of glandular odontogenic cyst requires at least some of these criteria: variable thickness of the epithelium lining the cyst, presence of hobnail cells, intraepithelial microcysts, clear cells in the basal and parabasal layers, papillary projections into the lumen and mucous cells. 10 With regard to the diagnosis of a dentigerous cyst, the epithelium should be enveloping the crown and be attached to the cervical region of an unerupted tooth. 10

It is important to reinforce that the mandibular cystic lesions composed exclusively of respiratory epithelium are rare. 1 2 Then, for diagnosis of mandibular surgical ciliated cysts, it is essential to establish the association of clinical/imagological and histopathological features with the patient' past history showing evidence of previous surgery in the mandible concomitant with the maxilla, as illustrated in our Brazilian patient.
